# Structural basis for glucosylsucrose synthesis by a member of the α-1,2-glucosyltransferase family: Crystal structure of a α-1,2-glucosyltransferase

**DOI:** 10.3724/abbs.2022034

**Published:** 2022-04-01

**Authors:** Qiuyu Han, Yuan Yao, Yuhan Liu, Wenlu Zhang, Jinyi Yu, Heya Na, Tianhao Liu, Kevin H. Mayo, Jiyong Su

**Affiliations:** 1 Engineering Research Center of Glycoconjugates Ministry of Education Jilin Provincial Key Laboratory of Chemistry and Biology of Changbai Mountain Natural Drugs School of Life Sciences Northeast Normal University Changchun 130024 China; 2 Media Academy Jilin Engineering Normal University Changchun 130052 China; 3 Department of Biochemistry Molecular Biology & Biophysics University of Minnesota Minneapolis MN 55455 USA

**Keywords:** catalytic mechanism, crystal structure, glucosylsucrose, UDP-glucose, α-1,2-glucosyltransferase

## Abstract

Glucosylsucroses are potentially useful as additives in cosmetic and pharmaceutical formulations. Although enzymatic synthesis of glucosylsucroses is the most efficient method for their production, the key enzyme that produces them has remained unknown. Here, we report that glucosylsucrose synthase from *Thermosynechococcus elongatus* (TeGSS) catalyzes the synthesis of glucosylsucrose using sucrose and UDP-glucose as substrates. These saccharides are homologous to glucosylsucroses produced by *Nostoc* sp. PCC 7120 (referred to as protein alr1000). When the ratio of UDP-glucose to sucrose is relatively high, TeGSS from cyanobacteria can hydrolyze excess UDP-glucose to UDP and glucose, indicating that sucrose provides a feedback mechanism for the control of glucosylsucrose synthesis. In the present study, we solved the crystal structure of TeGSS bound to UDP and sucrose. Our structure shows that the catalytic site contains a circular region that may allow glucosylsucroses with a right-hand helical structure to enter the catalytic site. Because active site residues Tyr18 and Arg179 are proximal to UDP and sucrose, we mutate these residues ( *i.e*., Y18F and R179A) and show that they exhibit very low activity, supporting their role as catalytic groups. Overall, our study provides insight into the catalytic mechanism of TeGSS.

## Introduction

Cyanobacteria can exist in very harsh environments, such as in hot springs, hypersaline water, deserts, and polar regions [1]. When cyanobacteria are under abiotic stress, they synthesize various compounds including sucrose, trehalose, glucosylglycerol, glycine betaine, *etc*. that act to stabilize their proteins and membranes [ 2, 3]. Thus such carbohydrate molecules have significant potential as food additives, cosmetic supplements, and pharmaceutical formulations [ 4– 6]. Although glucosylsucrose (also termed fru-oligosaccharides, sucroglucans or α-1,2 glucans) was identified in cyanobacteria in the 1960s [ 7, 8], the key enzyme involved in its enzymatic synthesis has yet to be identified.

NMR spectroscopy [ 7– 10] and mass spectrometry [11] have been used to elucidate the chemical structure of glucosylsucrose in which sucrose is covalently linked to the reducing-end of the molecule via an α-1,2 glycosidic bond. This motif suggests that sucrose may be a “primer” for the synthesis of glucosylsucrose in a similar fashion to the production of “short glycogen chains” formed during the initiation of glycogenesis [12]. The structure of glucosylsucrose suggests a catalytic mechanism, *i.e*., UDP-glucose+sucrose/glucosylsucrose→oligo-(1→2)-α-D-glucopyranosyl-(1→2)-β- *D*-fructofuranosides + UDP. In fact, Horacio et al. [7] tested this hypothesis and found that toluene-permeabilized cells of *Anabaena* sp. PCC 7120/ *Nostoc* sp. PCC 7120 can synthesize glucosylsucrose by using UDP-[ ^14^C]Glc and sucrose as substrates [7]. Therefore, the actual name for this enzyme should be UDP-glucose:sucrose/glucosylsucrose α-1,2-glucosyltransferase. To date, only three enzymes (ALG10, DSR-E and WaaJ) have been reported to catalyze the synthesis of an α-1,2 glycosidic bond [ 13– 16]. The proposed catalytic mechanism for the enzyme TeGSS is different from those of ALG10, DSR-E and WaaJ. In this regard, UDP-glucose:sucrose/glucosylsucrose α-1,2-glucosyltransferase is a novel enzyme.

NMR studies also showed that glucosylsucroses have a strong tendency to form right-handed helical structures, with the plane of the fructofuranose ring at the reducing-end of glucosylsucrose running perpendicular to the plane of the pyranose rings of the right-handed helix [8]. This structure is distinct from that in glycogen [17], starch [18], cellulose [13], as well as α-1,3 glucans [ 3, 14, 15] and β-1,3 glucans [16]. Due to these structural differences, glucosylsucrose may have some different biological functions that need to be elucidated. In the present study, our priority was to investigate how UDP-glucose:sucrose/glucosylsucrose α-1,2-glucosyltransferase sythnesizes glucosylsucrose.

When cyanobacteria are under salt- and heat-induced stress, they can synthesize about 4 to 20 times of the amount of sucrose and glucosylsucroses compared to normal conditions [ 10, 19]. Sucrose phosphate synthase (SPS) is the rate-limiting step in the enzymatic synthesis of sucrose [20], and stimulation of SPS activity can lead to sucrose accumulation in the cell and help cyanobacteria adapt to high saline conditions [ 21– 23]. Regulation of SPS activity and its catalytic mechanism have been extensively studied [ 24– 26]. However, little is known about the enzyme that synthesizes glucosylsucrose or how this synthesis relates to that of sucrose.

Recently, we identified SPS (TeSPS, Uniprot protein code: tll1590) from *Thermosynechococcus elongatus* [25]. In the *T*. *elongatus* genome, the *tll1591* gene (a possible glycosyltransferase) is proximal to the *tll1590* gene. In the present study, we used thin layer chromatography (TLC) to investigate the catalytic activity of tll1591. Our results show that this enzyme can transfer glucose from UDP-glucose to sucrose and form longer oligosaccharides. Moreover, glucosylsucrose synthesized by tll1591 is essentially the same as carbohydrates purified from *Nostoc* sp. PCC 7120 under saline-induced stress. Therefore, tll1591 is an UDP-glucose:sucrose/glucosylsucrose α-1,2-glucosyltransferase, prompting us refer to the *tll1591* gene as *Thermosynechococcus elongatus* glucosylsucrose synthase (TeGSS). Here, we also solved the crystal structure of TeGSS bound to UDP and sucrose, thus providing insight into the catalytic mechanism of the enzyme.

## Material and Methods

### Cloning, protein expression, and purification

The genes for *tll1591* and a *lr1000* were synthesized by SynBio Technologies (Monmouth Junction, USA), and cloned into pET28a vectors (Novagen, Gibbstown, USA). Site-directed mutagenesis of *tll1591* followed the manual of the QuickChange XL Site-directed Mutagenesis kit (Stratagene, La Jolla, Canada). All constructs were checked by DNA sequencing. The tll1591 construct, its mutants, as well as the alr1000 construct, were transfected into *E*. *coli* BL21 (DE3) cells and plated onto LB agar plates supplemented with 100 μg/mL kanamycin. Following overnight culturing, *E*. *coli* BL21 (DE3) cells were scraped from the agar plates and transferred into 10 mL of LB media containing 100 μg/mL kanamycin. This culture was then shaken at 37 ^o^C for 16 h. The following day, the LB medium containing *E*. *coli* BL21 (DE3) cells were transferred into 1 L of LB medium and shaken at 37 ^o^C. When the optical density of cultures reached 1.2–1.5, IPTG (final concentration of 0.5 mM) was added to induce protein over-expression. After overnight induction at 37 ^o^C, cells were harvested by centrifugation (6000 *g* for 15 min) and lysed by sonification in a lysis buffer that consisted of 10 mM Tris/HCl, pH 8.0, 150 mM NaCl, and 20 mM imidazole. The protein from the clarified cell extract was purified using a Ni-NTA agarose column (Qiagen, Hilden, Germany). After purification, the His-tagged protein was dialyzed in 10 mM Tris/HCl, pH 7.5, 150 mM NaCl, with thrombin (20 NIH units per milligram of protein) added to remove the His tag. SDS-PAGE demonstrated that protein purity was >90%. Proteins were concentrated to approximately 20–30 mg/mL and stored at −80°C.

### Crystallization, data collection, and structure determination

Crystals of the tll1591 gene product were obtained after 7 and 14 days from hanging drops that contained 1 μL of 10–20 μg/μL protein and 1 μL solution containing 1.5 M ammonium sulfate, 50 mM MES, pH 5.5, 10% (v/v) 1,4-dioxane, 5 mM UDP (for the 7FGA structure) and 1.1 M NaH _2_PO _4_, 0.28 M K _2_HPO _4_, 5 mM UDP (for the 7FG9 structure) at room temperature. Prior to X-ray data collection, crystals from the first condition were soaked for 5 min in the reservoir solution supplemented with 50% (v/v) saturated sucrose. For the second condition, 20% (v/v) glycerol was used as a cryoprotectant. Crystals were flash cooled in liquid nitrogen, and data sets were collected at 100K at the Shanghai Synchrotron Radiation Facility (Shanghai, China).

Data sets were indexed and integrated using the programs XDS [27] or iMosflm [28], and scaled using Aimless [29] from the CCP4 software package [30]. Structures were determined using Phaser [31] and molecular replacement with the structure of an UDP glucose α-tetrahydrobiopterin glycosyltransferase (PDB:5ZFK) as the search model. Structure refinement and water updating were performed using Phenix [32] refine and manual adjustment. Final structure validations were performed using MolProbity [ 33, 34], and figures of all structures were generated using Pymol ( https://pymol.org/2/).

### Thin layer chromatography (TLC)

A 10 μL reaction vessel containing TeGSS in a solution of 10 mM Tris HCl, pH 7.5, 10 mM sucrose, 10 mM UDP-glucose (UDPG), 2 μg enzyme (5 μM in 10 μL) was prepared and run at 40 ^o^C for 1 h or overnight. The reaction was stopped by dropping the temperature to –20 ^o^C. A glass capillary was used to draw up 3–5 μL of the reaction solution that was placed on the same silica gel thin-layer plate and developed using n-butanol-acetone-water (4:3:1), and then dried using 2% aniline acetone solution [2% diphenylamine acetone solution; 85% phosphoric acid (v:v:v=5:5:1)] as the color developing agent. The plate was heated using a hair dryer until the spots became clear. The effects of pH, temperature, substrate ratio, and point mutations, on enzyme activity, were investigated.

### Extraction of glucosylsucroses from *Nostoc* sp. PCC 7120

*Nostoc* sp. PCC 7120 was grown in BG11 medium at 25–30 ^o^C on a windowsill. A total of 1 L BG11 medium contained 17.6 mM KNO _3_, 0.175 mM K _2_HPO _4_, 1.3 mM MgSO _4_, 0.25 mM CaCl _2_, 0.028 mM citric acid, 0.028 mM ferric citrate, 0.0034 mM EDTA, 0.19 mM Na _2_CO _3_ and 1 mL of trace element solution (61.0 mg H _3_BO _4_, 169 mg MnSO _4_·7H _2_O, 287 mg ZnSO _4_·7H _2_O, 2.5 mg CuSO _4_·5H _2_O, and 12.5 mg (NH _4_) _6_MoO _24_·4H _2_O in 1 L water). After one week of growth, 80 mM NaCl [19] was added to the culture to stress *Nostoc* sp. PCC 7120 for one day. Afterwards, *Nostoc sp*. PCC 7120 was harvested by centrifugation (12,000 rpm for 10 min). Approximately 0.2 g *Nostoc* sp. PCC 7120 pellet could be harvested from 30 mL of culture. After most of the BG11 medium was discarded, about 30 μL of BG11 medium remained in the *Nostoc* sp. PCC 7120 pellet which was heated at 100 ^o^C for 10 min. Extracts were separated from the precipitate by centrifugation (12000 rpm for 10 min), and glucosylsucrose in the extracts was assessed by using TLC.

### Coupled assay to determine UDP

The kit (Item No. BC3330) for detecting UDP was purchased from Solarbio (Bejing, China). The assay followed instructions provided with the manufacturer’s kit that contained NADH, pyruvate kinase, and lactate dehydrogenase. The two enzymes catalyzed the oxidation of NADH to NAD ^+^, and the rate of NADH conversion was measured at 340 nm to reflect TeGSS activity.

### High performance liquid chromatography (HPLC)

Before HPLC analysis, a 30-μL reaction vessel containing a solution of 2.5 μM TeGSS (or mutants Y18F or R179A), 30 mM sucrose and 30 mM UDP-glucose, was incubated for 1 h or overnight at 40 ^o^C. Samples were then centrifuged for 15 min at 12,000 rpm at 4 ^o^C before loading onto the HPLC system (LC-16; Shimadzu Corporation, Tokyo, Japan)-RID (RID-20A; Shimadzu Corporation, Tokyo, Japan) with a polymeric amino column (NH2P-50 4E; Shodex, Tokyo, Japan) to analyze the glucosylsucroses synthesized by TeGSS and mutants (Y18F and R179A). The flow rate was 1 mL/min, and the operational temperature was set at 30 ^o^C. The injection volume was 10 μL, and an isocratic elution was performed as the mobile phase with 65% chromatography grade acetonitrile filtered with a PVDF filter of 0.45 μm.

## Results

### Crystal structure of TeGSS bound to UDP and sucrose

Initially, we purified TeGSS from *E*. *coli* BL21 (DE3) for crystallization and enzymatic analysis ( Supplementary Figure S1). When we first attempted to crystallize the enzyme, we had no idea that TeGSS is a UDP-glucose:sucrose/glucosylsucrose α-1,2-glucosyltransferase, and thus our initial crystallization attempts failed. The failure arose because the presence of substrate or product is crucial to stabilizing the overall structure of the enzyme. When we finally realized this (see enzymatic assay section), we added UDP to the crystallization buffers and soaked the resulting crystals with sucrose. This allowed us to produce the crystals and solve the structures of TeGSS.

**Figure 1:**
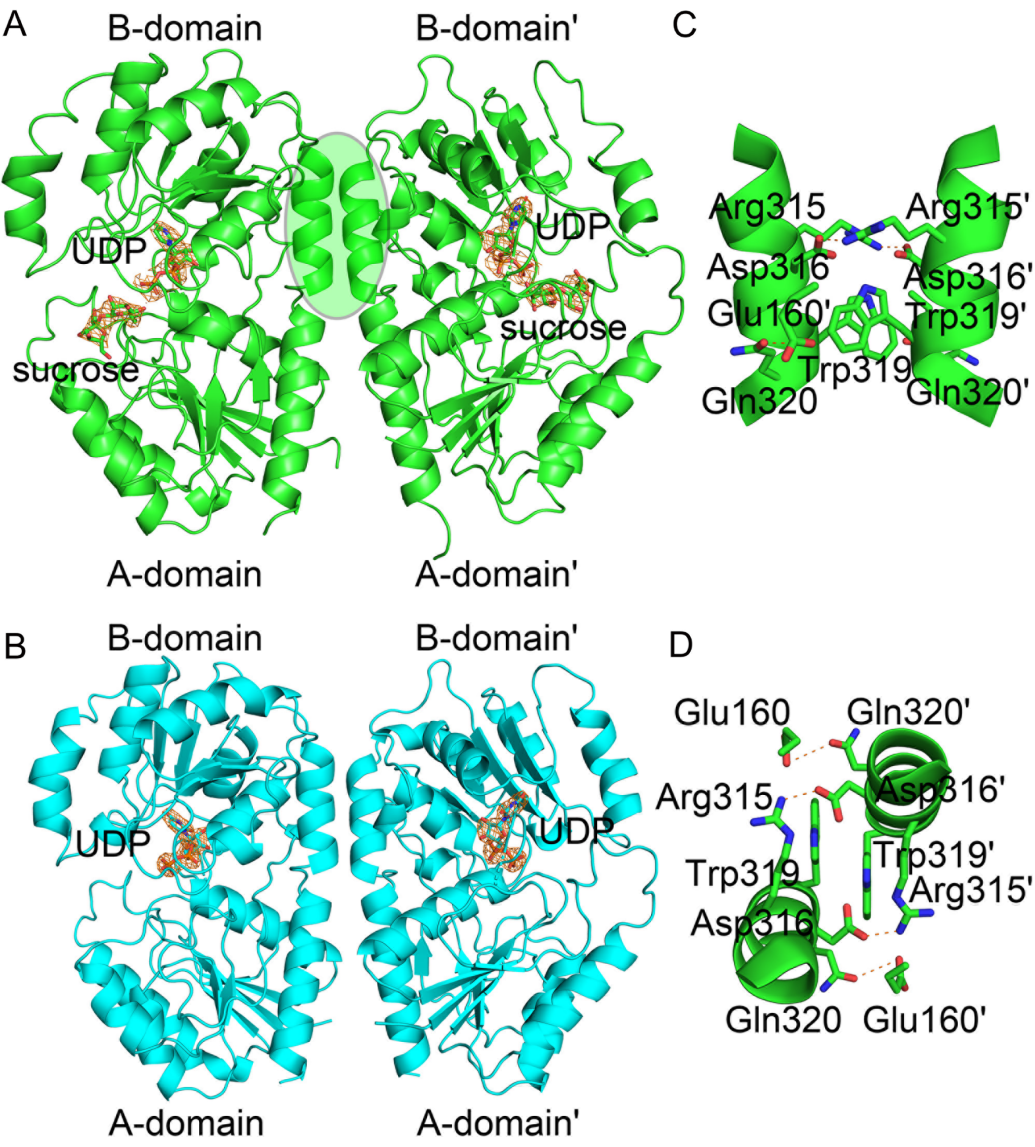
Figure1 Structure of TeGSS (A) Crystal structure of TeGSS bound to UDP and sucrose. TeGSS has two Rossmann-type folds, termed A- and B-domains. The 2|Fo|-|Fc|, αc map contoured at 1.0δ of UDP and sucrose is shown in gold. TeGSS crystallized as a homodimer. The interaction domain formed by two TeGSS monomer subunits is indicated by the green oval. (B) Crystal structure of TeGSS bound to UDP. The 2|Fo|-|Fc|, αc map contoured at 1.0δ of UDP is shown in gold. (C,D) The top and side views of the interaction domain between two TeGSS monomer subunits is shown, and the ionic, hydrogen, and π-π bonds stabilize the TeGSS dimer structure.

In the first structure, TeGSS was bound with UDP at the interface of the B-domain, with sucrose loosely interacting with the A-domain ( Figure 1 and Supplementary Figure S2). In the second structure, TeGSS was found to only bind to UDP ( Figure 1 and Supplementary Figure S2). RMSD values for these two structures fell between 0.292 and 0.633, indicating that both structures have similar global folds. Both structures crystallized as dimers ( Figure 1A,B, overall structures), with structural statistics provided in Table 1. In particular, five residues (Glu160, Arg315, Asp316, Trp319 and Gln320) stabilize the dimer structure ( Figure 1C,D). The guanidinium group of Arg315 from one monomer forms an ionic bond with the carboxyl group of Asp316 from another monomer, and the indole side chains of two Trp319 residues from both monomers interact with each other through a π-π bond. Furthermore, the carboxyl group of Glu160 from one monomer subunit forms a hydrogen bond with the Gln320 side chain of another subunit. Overall, the TeGSS dimer is stabilized by non-covalent bonds.

**Table:** **Table1** Data collection and refinement statistics

PDB code	7FGA	7FG9
Resolution (Å)	19.96-3.00 (3.27-3.20)	19.23-2.66 (2.79-2.66)
Space group	P6 _5_	P6 _3_
Unit cell parameters (a, b, c) (Å), (α, β, γ) (°)	(157.71, 157.71, 187.80), (90.00, 90.00, 120.00)	(177.64, 177.64, 57.03),(90.00, 90.00, 120.00)
No. of measured reflections	251286 (34251)	124732 (16014)
No. of unique reflections	43413 (6364)	28981 (3875)
Completeness (%)	99.4 (100.0)	98.1 (99.3)
Multiplicity	5.8 (5.4)	4.3 (4.1)
*R*_merge_ (%)	19.0 (99.1)	7.3 (50.5)
< *I*/δ( *I*)>	6.3 (1.6)	12.8 (2.5)
*R*_model_ (%)	29.6	17.85
*R*_free_ (%)	32.6	22.65
Rmsd bond lengths (Å)	0.002	0.01
Rmsd bond angles (°)	0.80	1.281
Ramachandran plot ^f^ residues in favored regions (%)	90	95
Substrate/ligand	UDP, sucrose	UDP

Our crystal structures indicate that TeGSS is a GT-B type enzyme [35] that has two domains: A and B ( Figure 1A,B). The overall structure of TeGSS is highly similar to that of other GT-B type glycosyltransferases, *i.e.*, TeSPS [25], *H*. *orenii* SPS [26], *A*. *tumefacients* glycogen synthase [36], *E*. *coli* OtsA [37]. All of them have a dumbbell-shaped structure, with only two loops connecting the A- and B-domains, suggesting that glycosyltransferases may adopt different conformations. In fact, glycogen synthase from *A*. *tumefacients* was crystallized in an open conformation, and OtsA from *E*. *coli* was crystallized in a closed conformation [ 36, 37]. Here, TeGSS adopts a closed conformational state in both of our structures.

The catalytic core of TeGSS is located at the interface of the A- and B-domains ( Figure 1A,B). When TeGSS is in an open conformational state, the A- and B-domains can freely move in solution, and the catalytic cavity cannot form. In contrast, when TeGSS binds to substrates or products, TeGSS is transitioned by induced fit into the closed conformational state that then stabilizes the catalytic site. Our two structures of TeGSS are bound with UDP; thus the presence or absence of sucrose in the catalytic site does not significantly influence the global structure of the enzyme. This indicates that UDP is key to stabilizing the closed conformation, as opposed to sucrose or longer glucosylsucroses. The binding of UDP to TeGSS suggests that saccharide products can escape from the catalytic center prior to the release of the UDP by-product.

### The catalytic core

**Figure 2:**
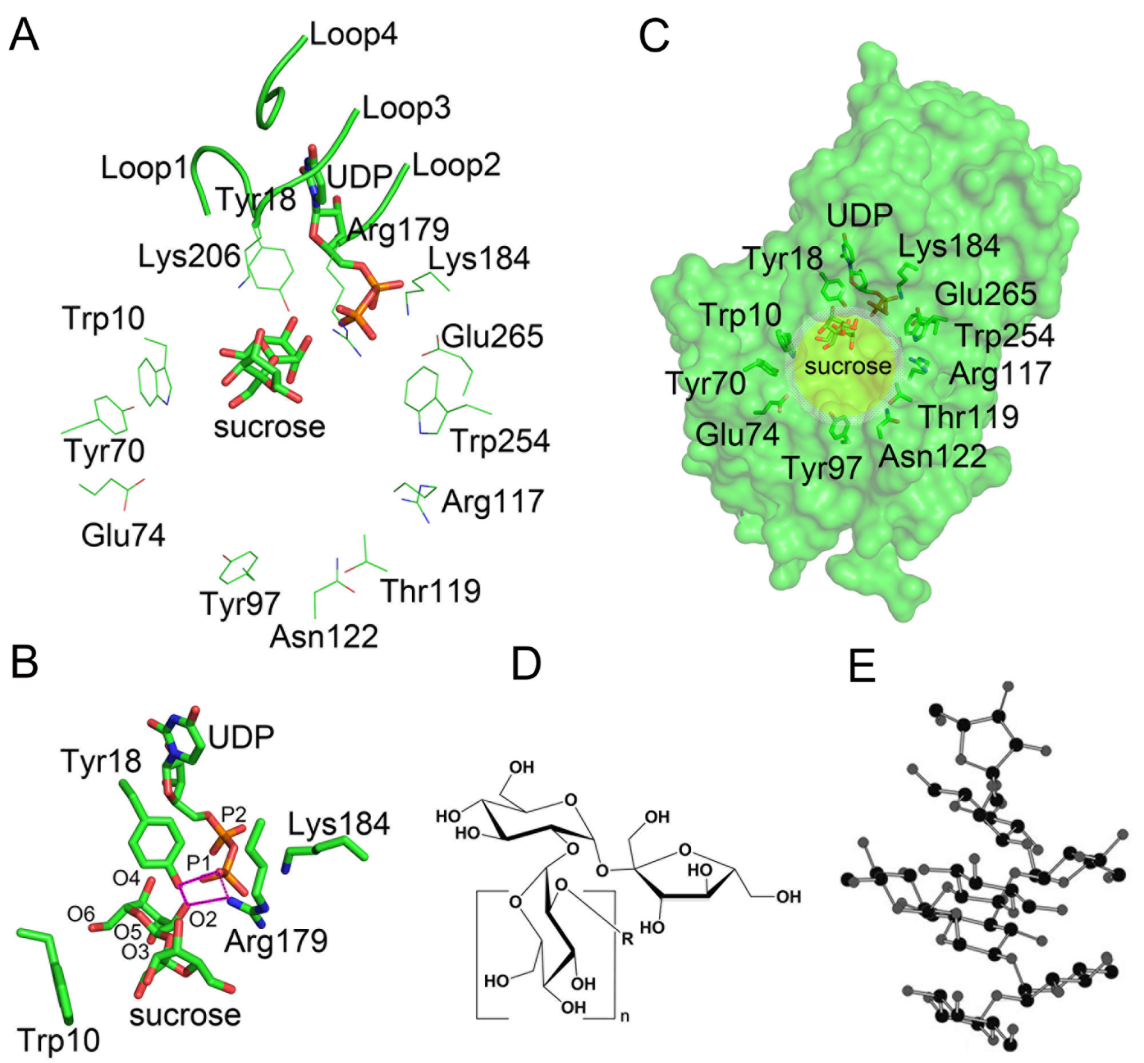
Figure2 Catalytic center of TeGSS (A) UDP and sucrose were co-crystallized at the TeGSS catalytic center. Four loops stabilize the uridine group in UDP, with Tyr18, Arg179, Lys184 and Glu265 residues stabilizing the diphosphate group of UDP. Sucrose is loosely bound within the catalytic center. (B) Trp10 forms a weak π-π bond with the fructose residue from sucrose. Tyr18 and Arg179 simultaneously interact with the O2 atom of sucrose and the P1 phosphate group of UDP, indicating that these residues directly participate in the catalytic reaction. (C) Eleven peripheral residues within the catalytic center form a circular ridge that may regulate TeGSS activity by recognizing suitable substrates. (D) The general molecular formula of glucosylsucrose. (E) Glucosylsucrose [8] can form a right-hand helix that may be able to fit in the catalytic center of TeGSS.

Within the catalytic core, the uridine group of UDP is trapped by four loops 1, 2, 3 and 4 (loop 1: Pro15–Tyr18; loop 2: Leu177–Arg179; loop 3: Ala203–Val206; and loop 4: Ile230–Asp234) ( Figure 2A). The diphosphate group of UDP forms hydrogen or ionic bonds with Tyr18, Arg179, Lys184 and Glu256 ( Figure 2A). Therefore, UDP can bind stably to the catalytic center. In contrast, sucrose interacts only weakly with the catalytic core. During co-crystallization, we soaked TeGSS crystals in 50% saturated sucrose solution to solve the co-crystal structure of TeGSS with UDP and sucrose bound. In the catalytic center, only three amino acid residues form non-covalent bonds with sucrose. Trp10 forms a weak π-π bond with the fructose residue of sucrose, and the hydroxyl group of Tyr18 and the guanidinium group of Arg179 form hydrogen bonds with the glucose residue of sucrose ( Figure 2B). The advantage of this loose binding state is beneficial for product departure, as well as to accommodate different sizes of glucosylsucrose substrates. However, this low affinity can influence the catalytic efficiency of TeGSS.

The reaction center contains many acidic and basic residues that surround UDP and sucrose. These residues may constitute a charge relay network that could directly participate in catalysis ( Figure 2A). In this instance, Arg179 forms an ionic bond with the terminal phosphate group (P1) of UDP ( Figure 2B). In turn, this phosphate group is also proximal to the glucose residue of sucrose, implying that Arg179 plays a role in catalysis. In fact, our R179A mutant shows very low activity compared with wild-type TeGSS. In addition to the charged amino acid residues, Tyr18 is very close to UDP and sucrose and effectively forms a triangle with both molecules ( Figure 2B). Here, we propose that following UDP-glucose binding to TeGSS, Tyr18 participates in forming a transition state with UDP-glucose. In our enzyme assays, the Y18F mutant was only minimally active towards these substrates.

In the closed conformational state, eleven residues (Trp10, Tyr18, Tyr70, Glu74, Tyr97, Arg117, Thr119, Asn122, Lys184, Trp254 and Glu265) at the surface of TeGSS lie at the edge of the catalytic cavity, the diameter of which is approximately 15 Å ( Figure 2C). Glucosylsucroses can spontaneously form a right-hand helix ( Figure 2D,E) [8], with their diameters being less than 15 Å. In this regards, this circular cavity may promote entry of glucosylsucrose. Overall, the circular catalytic cavity may help TeGSS select substrates and promote the catalytic reaction.

### Catalytic activity of TeGSS

At the start of our study, we used on-line databases ( www.brenda-enzymes.org, https://www.ncbi.nlm.nih.gov/cdd/, and www.cazy.org) to predict the class of enzyme to which TeGSS belongs. However, the knowledge that most glycosyltransferases normally have two kinds of substrates made it difficult for us to determine the class. Since the gene of TeGSS/tll1591 is proximal to the TeSPS/tll1590 gene that is an SPS, we speculated that TeSPS and TeGSS might constitute an operon that is regulated by the same stimuli. In addition, because TeSPS is a key enzyme for sucrose synthesis, we hypothesized that the reaction that TeGSS catalyzes is related to the synthesis of sucrose or at least involves sucrose.

**Figure 3:**
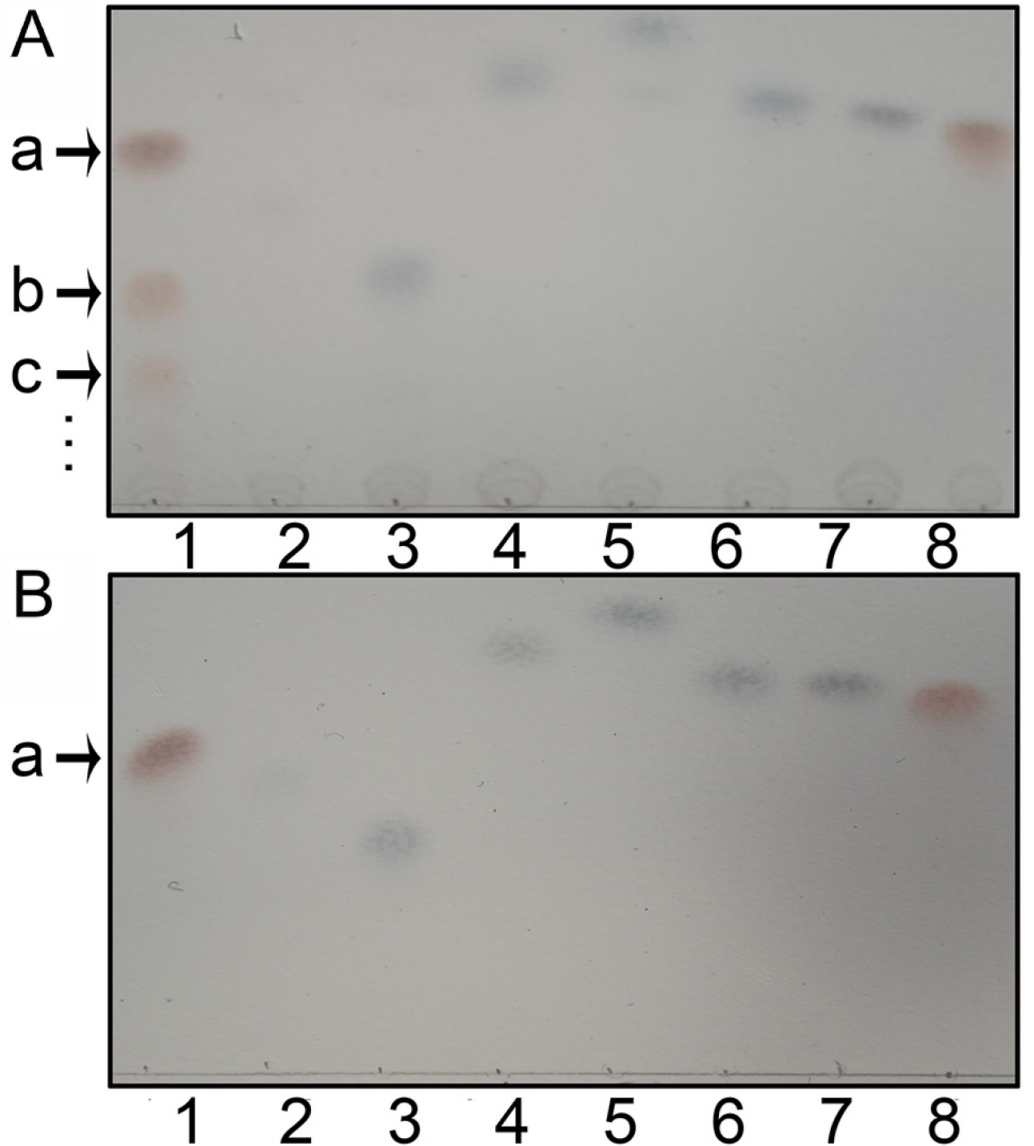
Figure3 Synthesis of glucosylsucroses by TeGSS The following letters identify various carbohydrate molecules: “a” for sucrose, “b” for trisaccharide, “c” for tetrasaccharide, and “…” for larger glucosylsucroses that cannot be separated by TLC. (A) TeGSS can synthesize glucosylsucroses only by using UDP-glucose and sucrose as substrates (lane 1). TeGSS cannot synthesize glucosylsucroses by using other monosaccharides or disaccharides, including maltose (lane 2), lactose (lane 3), mannose (lane 4), xylose (lane 5), arabinose (lane 6), glucose (lane 7), and fructose (lane 8). (B) TLC of all monosaccharides and disaccharides used in (A). Lane 1 for sucrose, lane 2 for maltose, lane 3 for lactose, lane 4 for mannose, lane 5 for xylose, lane 6 for arabinose, lane 7 for glucose, and lane 8 for fructose.

To validate this hypothesis, we designed a screening assay to determine the activity and substrate specificity of TeGSS. Initially, we incubated TeGSS with UDP-glucose and several monosaccharides and disaccharides, and then ran TLC to assess the products formed. On the TLC plate, however, we only observed large amounts of products in the sucrose lane, but not in the lanes of other saccharides, like glucose or maltose ( Figure 3). The first and second bands below sucrose should be tri- and tetra-saccharides. Overall, this experiment demonstrated that sucrose and UDP-glucose are substrates of TeGSS. Because the TLC assay can measure the amount of carbohydrate produced, we used an additional coupled assay to measure the change in UDP concentration. Our results show that TeGSS apparently generates more and more UDP over time ( Supplementary Figure S3).

**Figure 4:**
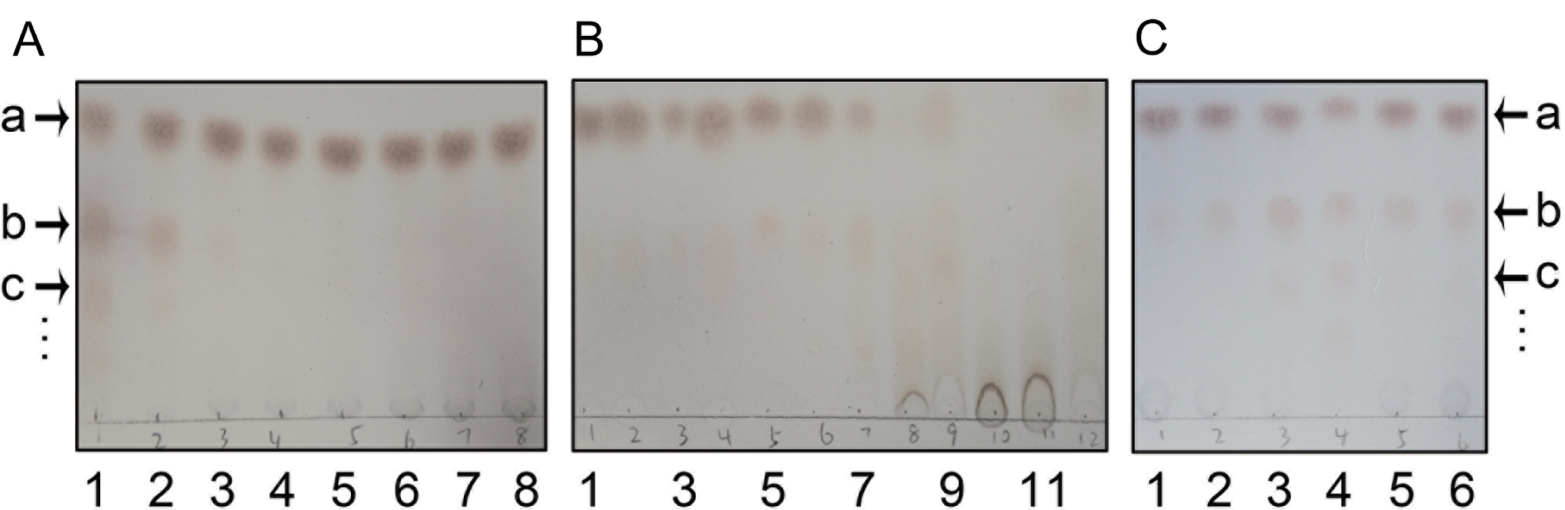
Figure4 Effects of enzyme concentration, pH, temperature, and ratio of UDP-glucose to sucrose on TeGSS activity The following letters identify various carbohydrate molecules: “a” for sucrose, “b” for trisaccharide, “c” for tetrasaccharide, and “…” for larger glucosylsucroses that cannot be separated by TLC. (A) Effect of enzyme concentration on TeGSS activity: lane 1 for the initial concentration of TeGSS (10 μg/μL), and lane 2 to lane 8 for a 1:1 serial dilutions of lane 1. (B) Effect of pH on TeGSS activity: lane 1 to lane 12 for pH values of pH 4.5 to pH 10.0. TeGSS has very good activity under basic solution conditions. (C) Effect of temperature on TeGSS activity: lanes 1, 2, 3, 4, 5 and 6 for 0, 20, 40, 60, 80, and 100oC, respectively. TeGSS exhibits its greatest activity at 60oC.

Next, we determined the effects of changes in enzyme concentration, pH, temperature, and the ratio of UDP-glucose and sucrose on TeGSS activity. First, we found that only when the enzyme concentration reached 10 μg/μL that glucosylsucroses were clearly observed on the TLC plate ( Figure 4A). Therefore, we used this TeGSS concentration in all further runs. Moreover, because 10 μg/μL is a relatively high concentration, it appears that TeGSS has a relative low efficiency *in vitro*. Secondly, although TeGSS can synthesize glucosylsucrose over a broad pH range from pH 4.5 to pH 10, the enzyme exhibits its highest activity between pH 9.0 and pH 9.5, and can also synthesize many higher molecular weight glucosylsucroses that could not be well separated on the TLC plate ( Figure 4B). We chose pH 8.0 as the best condition to test TeGSS activity, because this condition promotes synthesis of lower molecular weight glucosylsucroses, such as tri- and tetra-saccharides, which could be clearly separated by TLC. Subsequently, we found that the optimal temperature for the TeGSS reaction is 60°C, which is not surprising because this enzyme comes from a thermophilic bacterium ( Figure 4C).

**Figure 5:**
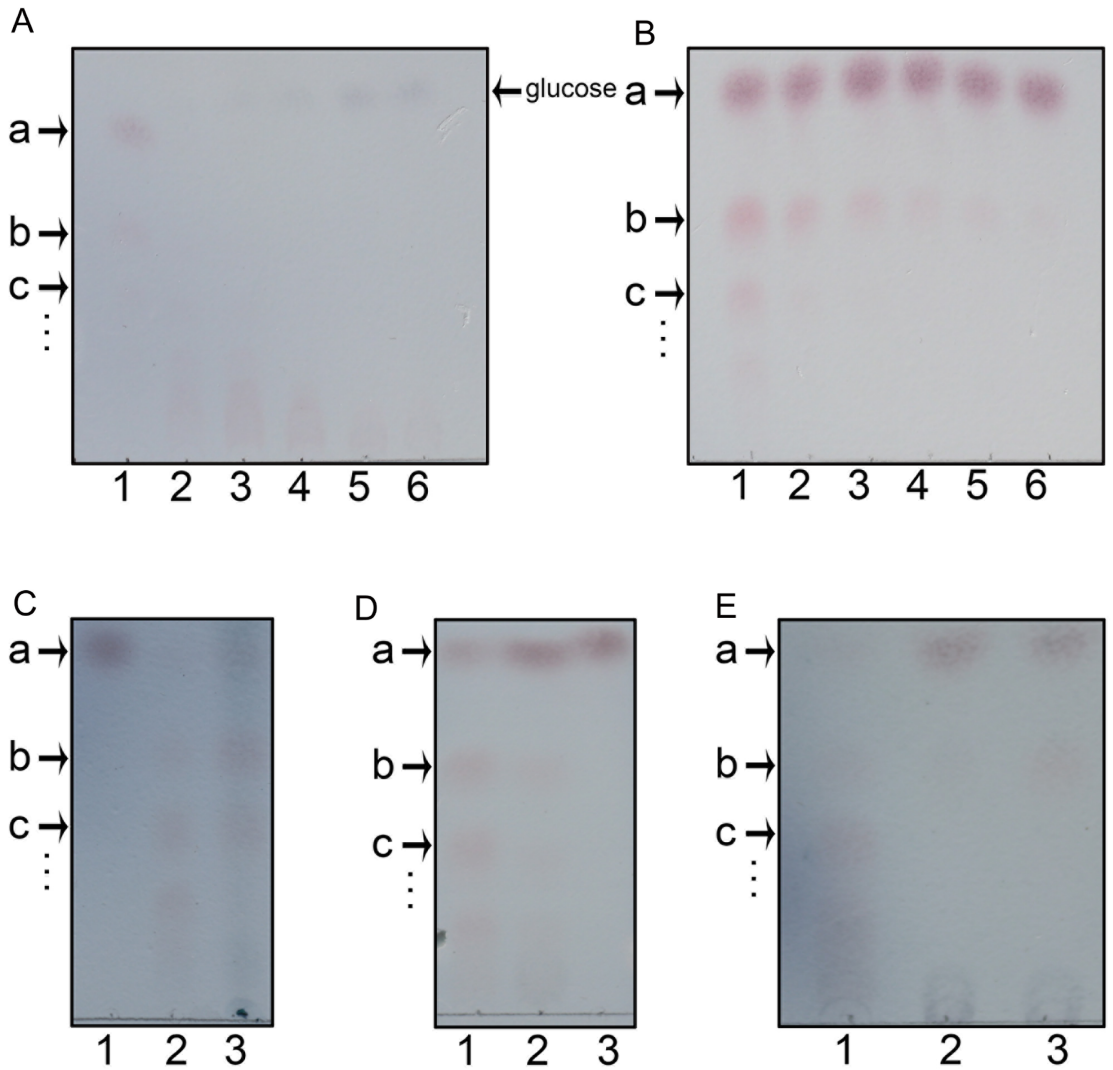
Figure5 Characterization of TeGSS and alr1000 The following letters identify various carbohydrate molecules: “a” for sucrose, “b” for trisaccharide, “c” for tetrasaccharide, and “…” for larger glucosylsucroses that cannot be separated by TLC. (A) Lane 1 is for 10 mM UDP-glucose and 10 mM sucrose used in the reaction. Lanes 2 to 6 are for 1:10 serial dilutions of the sucrose amount used in lane 1. With an increasing ratio of UDP-glucose to sucrose, TeGSS synthesizes larger molecular weight glucosylsucroses (see the faint pink bands at the bottom of the TLC plate). After TeGSS has consumed all of the sucrose present, it starts to hydrolyze UDP-glucose to UDP and glucose (See the faint blue bands at the top of TLC plate). (B) Lane 1 is for 10 mM UDP-glucose and 10 mM sucrose used in the reaction solution. Lanes 2 to 6 indicate 1:1 serial dilutions of UDP-glucose used in lane 1. When the molar ratio of UDP-glucose and sucrose is 1:1, TeGSS can synthesize large molecular weight glucosylsucroses, including tetra-saccharides, pentasaccharides, etc. In contrast, when the molar ratio of UDP-glucose to sucrose is lowered, TeGSS can only synthesize trisaccharides. (C) Lane 1 is for sucrose as a marker. Lane 2 is for TeGSS synthesis of glucosylsucroses. Lane 3 is for glucosylsucroses purified from saline-stressed Nostoc sp. PCC 7120. The migration of glucosylsucroses indicates that they are the same molecule and have the same molecular weight. (D) Lane 1 is for TeGSS synthesis of glucosylsucroses. Lane 2 indicates that alr1000 can also synthesize the same glucosylsucroses as TeGSS. Lane 3 shows sucrose as a marker. TeGSS and alr1000 belong to the class of enzyme that synthesizes glucosylsucroses. (E) Activity of mutants Y18F and R179A: lane 1 is for wild-type TeGSS that synthesizes glucosylsucroses. Y18F (lane 2) and R179A (lane 3) can only synthesize trisaccharides during an overnight reaction.

Finally, we determined the effect of the ratio of UDP-glucose to sucrose on the reaction, with the optimal ratio being 1:1. Because UDP-glucose and sucrose are both 10 mM, TeGSS can produce maximal amounts of tri- and tetra-saccharides. When the molar ratio of UDP-glucose to sucrose is high, TeGSS can quickly synthesize glucosylsucroses with higher molecular weights that cannot be separated well by TLC. Surprisingly, TeGSS could hydrolyze UDP-glucose to UDP and glucose after TeGSS synthesizes glucosylsucroses ( Figure 5A). However, when the concentration of sucrose was increased to 10 mM, TeGSS could only synthesize glucosylsucroses. This indicates that sucrose is the optimal substrate for TeGSS and implies that TeGSS can hydrolyze UDP-glucose when its concentration is too high or the concentration of sucrose is too low. This indicates that after TeGSS binds to UDP-glucose, UDP-glucose is transformed to a form that can readily loose glucose by transferring it to any nucleophilic group, such as the hydroxyl group of water or the oxygen anion at position 2 of glucose in sucrose.

Upon fixing the concentration of sucrose and examining the effect of different concentrations of UDP-glucose ( Figure 5B), we found that when the molar ratio of sucrose to UDP-glucose was high, only trisaccharides could be detected on the TLC plate. This indicates that TeGSS will use up all UDP-glucose to synthesize glucosylsucroses when a high concentration of sucrose is present in the cytoplasm of *Thermosynechococcus*.

When we discovered that TeGSS can transfer the glucose residue of UDP-glucose to sucrose, we purified glucosylsucroses from salt-stressed *Nostoc* sp. PCC 7120 [ 7, 10] and ran a TLC plate to compare the molecular weights of the enzyme-catalyzed glucosylsucroses and the glucosylsucroses extracted from *Nostoc* sp. PCC 7120. Our TLC results showed that the migration distances of both samples were exactly the same, as were the intensities of colored carbohydrate bands ( Figure 5C). This indicates that TeGSS is an UDP-glucose:sucrose/glucosylsucrose α-1,2-glucosyltransferase.

When we aligned the primary structure of TeGSS with that of *Nostoc* sp. PCC 7120 in NCBI blast ( https://blast.ncbi.nlm.nih.gov/Blast.cgi), we discovered that the alr1000 protein [38] had the greatest identity (63%) to TeGSS. The BLAST result also showed that many cyanobacterial proteins/enzymes are highly conserved with TeGSS, indicating that this kind of enzyme is widely distributed in cyanobacteria. By using the same purification procedure as with TeGSS, we obtained a large amount of alr1000 protein, and examined its activity. Following incubation of alr1000 with UDP-glucose and sucrose, our TLC results showed that the color and size of the glucosylsucrose synthesized by alr1000 is the same as that of TeGSS ( Figure 5D). This indicates that the alr1000 protein is also an UDP-glucose:sucrose/glucosylsucrose α-1,2-glucosyltransferase in *Nostoc* sp. PCC 7120.

In the co-crystal structure of TeGSS with UDP and sucrose, Tyr18 and Arg179 are proximal to both substrates, and thus may be crucial for its catalytic activity. Both residues are also highly conserved in this type of enzyme. To probe this, we employed site-directed mutagenesis to produce mutants Y18F and R179A. Our enzymatic assay showed that both mutants could not synthesize glucosylsucrose, even within 1 h. However, by running the reaction overnight, both mutants could synthesize small amounts of the trisaccharide ( Figure 5E). These results demonstrate that these two residues directly participate in catalysis.

**Figure 6:**
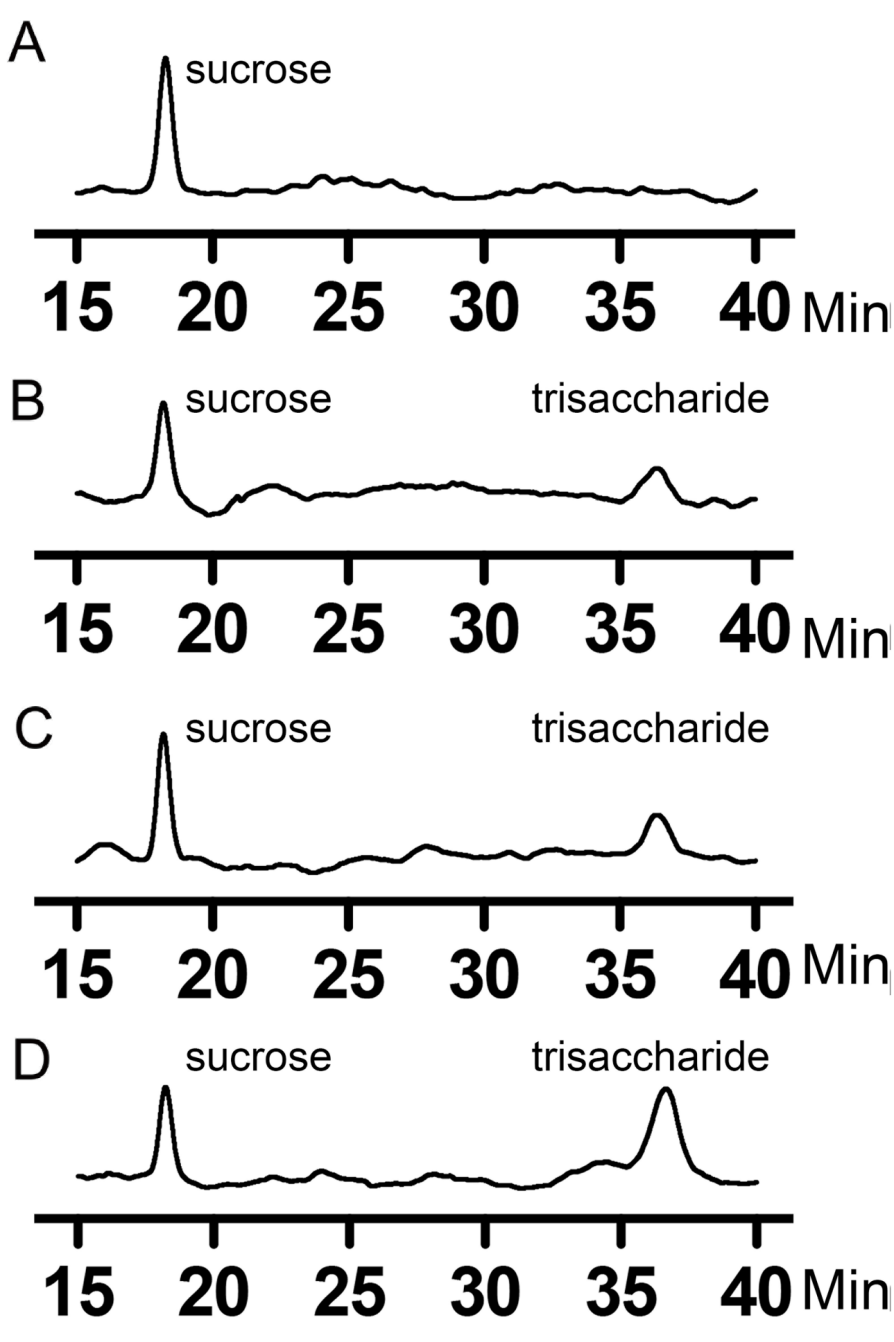
Figure6 HPLC of glucosylsucroses produced by the wild-type enzyme, and mutants Y18F and R179A (A) HPLC of sucrose. (B) HPLC of glucosylsucroses generated by wild-type enzyme (1 h of reaction). Aside from sucrose, the elution peak of the trisaccharide is identified. (C,D) HPLC of glucosylsucroses generated by Y18F and R179A (overnight reactions), demonstrating that Y18F and R179A can also synthesize trisaccharides.

Finally, we used HPLC to detect the presence of trisaccharide after the enzyme reaction. We only checked the amount of trisaccharide produced, because it is generally the largest one produced after an acceptable period of time ( Figures 5E). Sucrose was used as a control with an elution at 18.2 min ( Figure 6). After the TeGSS reaction, trisaccharide was detected with an elution at 36.4 min. Y18F and R179A also could synthesize the trisaccharide with the same elution volume as with wild-type enzyme ( Figure 6).

## Discussion

In glucosylsucroses, glucose is linked to sucrose via an α-1,2 bond [ 7, 10]. This structure is distinct from fructose-based polymers [39]. Nevertheless, the osmoregulatory function of plant fructose-based polymers led to the investigation of glucosylsucroses from cyanobacteria. Two independent groups reported that these glucosylsucroses are comparable, with their synthesis being greatly increased when cyanobateria are under saline or heat stress [ 10, 19]. Prior to our present study, the key enzyme required for the synthesis of glucosylsucrose remained a mystery. An initial study with *Nostoc* sp. PCC 7120 showed that an enzyme might catalyze sucrose and UDP-glucose to form glucosylsucrose [7]. In the present study, we directly demonstrated that TeGSS and alr1000 are the key enzymes for synthesis of glucosylsucroses. Both enzymes can transfer glucose from UDP-glucose to sucrose and synthesize oligomers that are equivalent to glucosylsucroses purified from saline-stressed *Nostoc* sp. PCC 7120. The sequence identity between TeGSS and alr1000 is relatively high at 63%, suggesting that TeGSS and alr1000 belong to a new family of enzymes.

When *Nostoc* sp. PCC 7120 is under saline stress, sucrose and glucosylsucroses are synthesized in large quantities [19]. This implies that the same activator can up-regulate the transcription of both SPS and alr1000. NtcA, a global nitrogen regulator in cyanobacteria, acts as a transcriptional activator of the encoding gene for SPS-B [40] and is also required to maintain high sucrose biosynthesis. Nevertheless, NtcA as the transcriptional activator of alr1000 needs to be verified. In addition, Nostoc sp. PCC 7120 is a filamentous cyanobacteria capable of nitrogen fixation in specialized cells (heterocysts) that terminally differentiate from vegetative cells under conditions of nitrogen starvation [41]. Two kinds of nitrogenases can be expressed in heterocysts [42]. Nitrogenase activity of a **Nostoc* sp *. PCC 7120 alr1000 mutant [38] was shown to be only capable of nitrogen fixation under anaerobic conditions [38]. Based on the results of this study, we infer that glucosylsucrose (the product of alr1000 activity) may assist *Nostoc* sp. PCC 7120 adapt to nitrogen starvation or help nitrogenase attain its full activity.

As a cyanobacterium compatible solute, sucrose should appear earlier than other substrates, including glucosylsucrose [23]. Comparison of the molecular weights of these two substrates suggests that sucrose is easier than glucosylsucrose to be synthesized, primarily because glucosylsucrose requires more energy and a greater carbon source for its synthesis. On the other hand, glucosylsucrose likely protects proteins and other biomolecules more effectively than sucrose. In addition, TeGSS can hydrolyze UDP-glucose when the ratio of UDP-glucose to sucrose is relatively high. This indicates that *in vivo*, the amount of sucrose can regulate synthesis of glucosylsucroses, implying that SPS indirectly regulates TeGSS activity.

**Figure 7:**
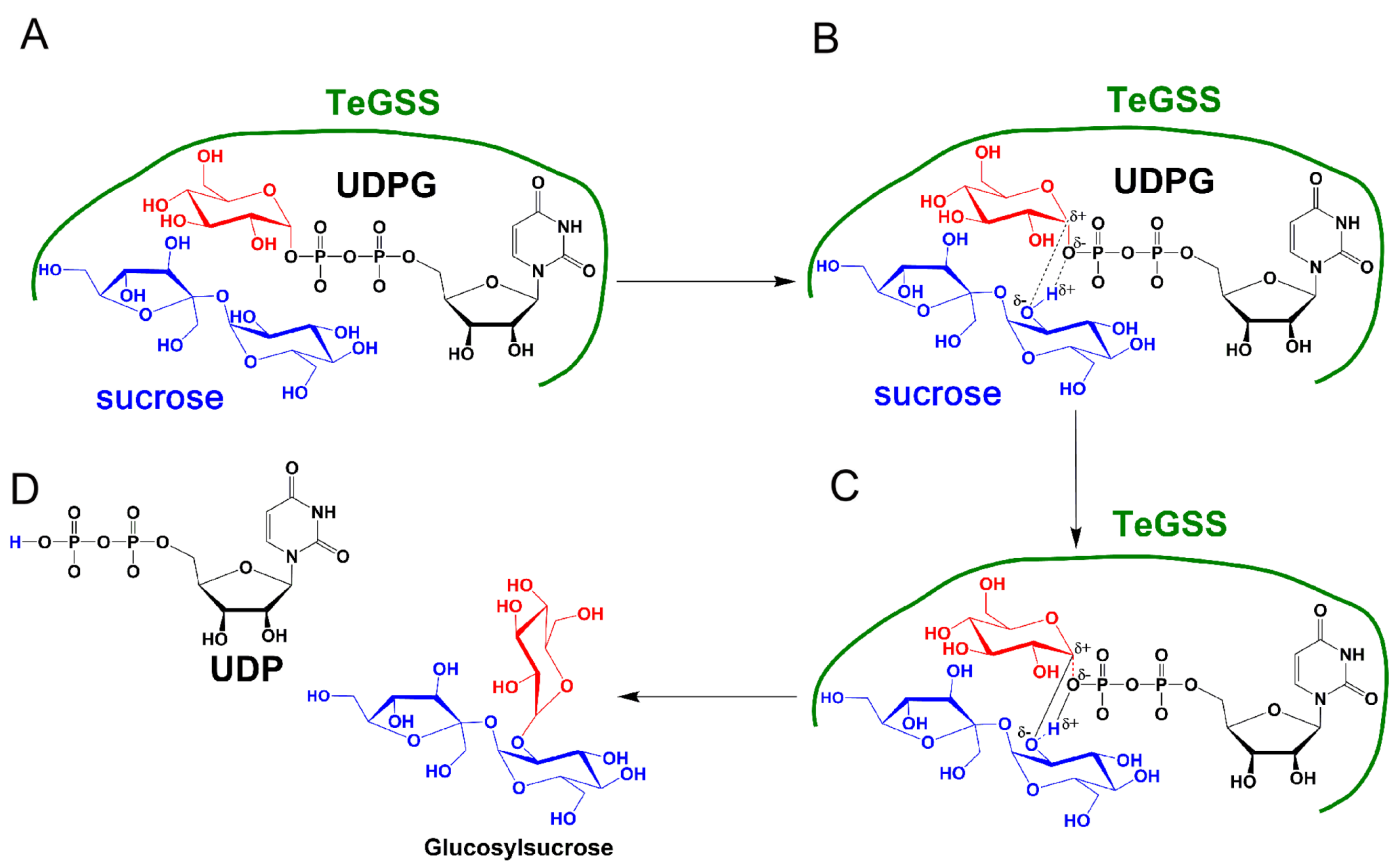
Figure7 Proposed catalytic steps with TeGSS (A) TeGSS first binds to sucrose and UDPG (UDP-glucose). (B) The amino acid residues in the TeGSS catalytic center induce sucrose and UDPG to form a transition state. C1 (δ+) of the glucose residue from UDPG is transformed into an oxocarbenium ion that may temporarily form an ionic bond with the C2 oxygen atom (δ-) of the glucose residue from sucrose. The oxygen atom (δ-) of UDPG phosphate forms a hydrogen bond with the hydrogen atom (δ+) linked to the C2 oxygen atom of the glucose residue from sucrose. (C) Simultaneously, two covalent bonds are formed during TeGSS catalysis. (D) Following catalysis, UDP and glucosylsucrose are released from the TeGSS catalytic center.

Glycosyltransferases catalyze the transfer of glycosyl groups to a nucleophilic acceptor, either by inversion or retention of configuration at the anomeric center. TeGSS transfers α-D-glucose from UDP-α-D-glucose to sucrose to form an α-1,2 glycosidic bond. Therefore, TeGSS is a retaining glycosyltransferase. There are several mechanisms that can be proposed to describe how glycosyltransferases catalyze their substrates [35]. For one, the Koshland retaining mechanism [43] appears reasonable, because it involves an enzymic nucleophile that can form a covalent intermediate with the glycosyl donor. In glycoside hydrolase families, a tyrosine residue at the catalytic center requires a general base to enhance its nucleophilic attack, which leads to formation of covalent intermediates with sugar residues [ 44– 46]. After this, the covalently-linked sugar residue can be transferred to a target molecular without changing its anomeric configuration. Originally, we thought that TeGSS likely follows this mechanism, because the requisite tyrosine (Try18) and arginine (Arg179) residues are present at the catalytic center. However, when we mutated these residues to Y18F and R179A, respectively, we observed that both can still synthesize glucosylsucroses, albeit minimally and only after overnight reactions. In any event, this suggests that TeGSS does not follow the Koshland retaining mechanism. Lee *et al*. [47] proposed an SNi catalytic mechanism to explain how another retaining glycosyltransferase OtsA functions. In this scheme, free energy relationships show that inhibitors of OtsA are synergistic transition state mimics, supporting a front-to-face nucleophilic attack mechanism involving hydrogen bonds forming between the leaving group (UDP-glucose) and the nucleophile. Kinetic isotope effects from the donor and acceptor substrates of OtsA have a highly dissociative oxocarbenium ion-like transition state [47]. Based on this model, we hypothesize that TeSPS (tll1590) also operates via an SNi catalytic mechanism, with residues within the catalytic cavity forming hydrogen bonds with UDP-glucose and promoting C1 of the glucose residue to form an oxocarbenium ion [25]. We also speculate that residues at the TeSPS catalytic center form hydrogen bonds (or other non-covalent bonds) with UDP-glucose and induce formation of a transition state that could be attacked by hydroxyl groups from water or glucose ( Figure 7). Although this mechanism for TeSPS is plausible, it does require further experimental validation.

In summary, upon abiotic stress, the estimated content of glucosylsucrose in cyanobacteria is between 0.25% and 0.5% (w/v) [10], and the total amount of cyanobacteria in the world is about 600 billion tons [48]. In other words, the total global amount of glucosylsucroses produced by cyanobacteria reaches about 1.5 to 3 billion tons/year. Therefore, studies on glucosylsucroses and the enzymes involved in their syntheses may have significant industrial and economic benefits. In the present study, we identified TeGSS and alr1000 as key enzymes in the synthesis of glucosylsucroses, and solved the crystal structure of TeGSS. Overall, this work provides crucial insight into enzyme-mediated glucosylsucrose synthesis.

## Supporting information

21551supplementary_Figures

## Supplementary Data

Supplementary data is available at *Acta Biochimica et Biophysica Sinica* online.
