# Supplementary material for: Structural basis for glucosylsucrose synthesis by a member of the α-1,2-glucosyltransferase family: Crystal structure of a α-1,2-glucosyltransferase
Source: Acta Biochim Biophys Sin (Shanghai). 2022 Apr 1;54(4):537–47. doi: 10.3724/abbs.2022034 (PMC9909042; doi:10.3724/abbs.2022034)
Supplement: 21551supplementary_Figures [file 21551supplementary_Figures.doc]

**Supplementary Figure S1. Purification of TeGSS**  The arrow indicates the purified TeGSS

**Supplementary Figure S2. Omit density maps.** The |Fo|-|Fc|, αc map contoured at 3.0δ of 7FGA structure (A) and of 7FG9 structure (B)

**Supplementary Figure S3. Assay measuring the amount of UDP**  When TeGSS is incubated with UDP-glucose for 15 minutes, no UDP is generated (flat solid square curve). In contrast, when TeGSS is incubated with UDP-glucose and sucrose, UDP appears (falling solid diamond curve).
